# Discovery of oxazine-linked pyrimidine as an inhibitor of breast cancer growth and metastasis by abrogating NF-κB activation

**DOI:** 10.3389/fonc.2024.1390992

**Published:** 2024-07-29

**Authors:** Jie Yuan, Bhanuprakash C. Narasimhachar, Akshay Ravish, Li Yang, Hua Zhang, Qun Wang, Zhi Li, Jun Huang, Bei Wang, Geng Wang, Keshav Kumar Harish, Arunachalam Chinnathambi, Chandramohan Govindasamy, Mahendra Madegowda, Basappa Basappa

**Affiliations:** ^1^ Department of Breast, Thyroid and Vascular Surgery, Hubei Provincial Clinical Research Center for Umbilical Cord Blood Hematopoietic Stem Cells, Taihe Hospital, Hubei University of Medicine, Shiyan, Hubei, China; ^2^ Department of Chemistry, Yuvaraja’s College, University of Mysore, Mysuru, Karnataka, India; ^3^ Laboratory of Chemical Biology, Department of Studies in Organic Chemistry, University of Mysore, Mysore, Karnataka, India; ^4^ Department of Clinical Laboratory Medicine, Taihe Hospital, Hubei University of Medicine, Shiyan, Hubei, China; ^5^ Department of Studies in Physics, University of Mysore, Mysore, India; ^6^ Department of Botany and Microbiology, College of Science, King Saud University, Riyadh, Saudi Arabia; ^7^ Department of Community Health Sciences, College of Applied Medical Sciences, King Saud University, Riyadh, Saudi Arabia

**Keywords:** breast cancer, nuclear factor-κB, cell viability and motility, TRX-01, oxazine linked pyrimidine, NF-κB activation, heterocyclic compounds, molecular orbital analysis

## Abstract

**Introduction:**

Nuclear factor kappa (NF-κB) plays a key role in cancer cell proliferation; thus, small molecule inhibitors of NF-κB activity can effectively inhibit breast cancer (BC) progression. We have previously reported oxazine and piperazine-linked pyrimidines as novel anti-cancer agents that can suppress NF-κB activation in BC cells. Moreover, the TRX-01 compound, an oxazine-linked pyrimidine, inhibited MCF-7 cells at a concentration of 9.17 µM in the Alamar Blue assay.

**Methods:**

This work involved the analysis of frontier molecular orbitals, HOMO-LUMO interactions, and molecular electrostatic potential for the TRX-01 structure. Additionally, the TRX-01 compound was studied for cytotoxicity, and migration as well as invasion assays were performed on BC cells.

**Results:**

Finally, TRX-01 blocked the translocation of NF-κB from the cytoplasm to the nucleus in MCF-7 cells and reduced NF-κB and IκBα levels in a dose-dependent manner. It also suppressed migratory and invasive properties of BC cells.

**Conclusion:**

Overall, the data indicates that TRX-01 can function as a novel blocker of BC growth and metastasis by targeting NF-κB activation.

## 1 Introduction

Cancer is a prominent cause of death and a significant barrier to life expectancy in every country. As the most frequent disease among women globally, breast cancer (BC) is expected to account for 43,000 deaths and over 290,000 new cases in the United States alone in 2022 (1, 2). The treatment landscape for breast cancer has undergone remarkable advancements over the past century, transitioning from predominantly surgical interventions to targeted therapies. Targeted therapies for breast cancer aim to identify new chemical compounds that hinder the progression of cancer cells by disrupting the biological pathways crucial for their growth and survival (3, 4). The detection of overexpression of specific receptors is a common occurrence in various types of BC. When these receptors are stimulated, they activate genes responsible for regulating tumor cell growth, migration, angiogenesis, and other crucial signaling pathways.

**Figure 1:**
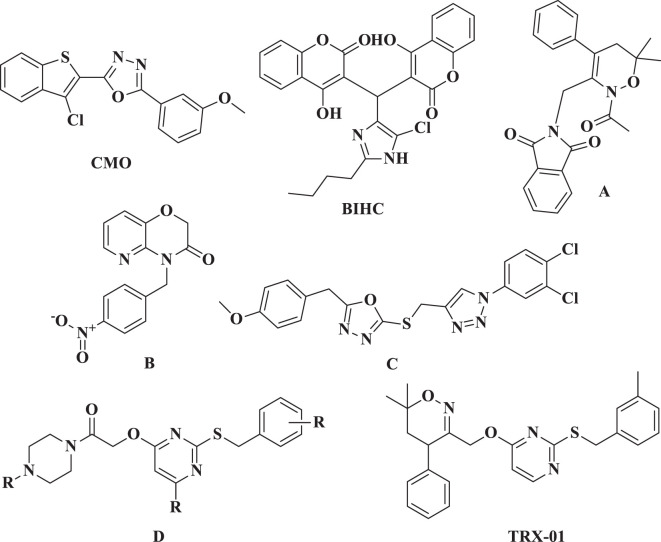
Previously reported NF-κB inhibitors.

Researchers have been focused on nitrogen-containing heterocycles for over a few decades because of their biological significance and diversity of structures (5, 6). We previously reported various heterocyclic compounds such as pyrimidine (7, 8), triazoles (9, 10), oxadiazoles (11–13), and many other heterocyclic entities that target various cellular pathways against cancer cells. Among other biological pathways, NF-κB has been recognized as an essential target for cancer treatment, particularly in the case of BC (14, 15). We have previously reported anti-cancer agents such as CMO, BIHC, A, B, C, D, and TRX-01 (**Figure 1**) that target NF-κB activation in various cancer cells (10, 16–19). Uncontrolled cell division may lead to cancer due to elevated activity of NF-κB. When NF-κB is activated, it can promote cell survival by blocking apoptosis. This activation influences the production of proteins that inhibit apoptosis, such as Bcl-2, Bcl-xL, and inhibitors of apoptosis proteins (IAPs), aiding cancer cells to avoid death and increasing their survival potential. Additionally, activation of NF-κB can enhance cell proliferation by increasing the expression of genes such as cyclins and cyclin-dependent kinases (CDKs) (20, 21).

In this work, we have provided a bioinformatics study of the compound of interest, TRX-01, and its effect on viability and motility of various BC cells. We have also investigated the influence of TRX-01 on the NF-κB signaling pathway in breast cancer cells, complemented by molecular dynamics studies.

## 2 Materials and methods

### 2.1 Materials

Dulbecco’s modified Eagle’s medium (DMEM), fetal bovine serum (FBS), rabbit secondary antibody, and mouse secondary antibody were obtained from Invitrogen (Carlsbad, CA). Thiazolyl blue tetrazolium bromide (MTT) and Dimethyl sulfoxide (DMSO) were purchased from Sigma-Aldrich (St. Louis, MO). Kits for DNA binding test and nuclear protein extraction were obtained from Active Motif Technologies (Carlsbad, CA). Cell Signaling Technology (Danvers, MA) provided the antibodies for p-NF-κB p65 (Ser536), NF-κB p65, p-IκBα (Ser32), IκBα, Lamin B, and β-actin antibodies.

### 2.2 Cell culture

Human MCF-7, MDA-MB-231, MDA-MB-468, SKBR3, T47D, BT474, BT549 and MCF-10A cells were obtained from ATCC. 10% FBS was added to DMEM for incubating the breast cancer cells. MCF-10A cells were cultured in a specialized medium (mammary epithelium basal medium, MEBM) containing 5% horse serum, 10 μg/ml insulin, 20 ng/ml epidermal growth factor, 100 ng/ml cholera toxin, and 0.5 μg/ml hydrocortisone. All cells were cultivated in a humidified 5% CO_2_ environment at 37°C.

### 2.3 Measurement of cell growth

The various cells were inoculated in 96-well plates at a rate of 5 × 10^3^ cells/well. 24 h later, cells were treated with different concentrations of TRX-01 (0–100 μM) for the indicated times. Subsequently, MTT assay was performed as described earlier (22).

### 2.4 Colony formation assay

2000 breast cancer cells per well were seeded in six-well plates. Following a 24-hour incubation period, cells were exposed to TRX-01 (0–100 μM) for a whole day. Then colony forming assay was done as described earlier (23). Colonies were fixed with 4% paraformaldehyde and counted by crystal violet staining.

### 2.5 Wound healing assay and invasion assay

The wound healing and invasion assays were conducted as described earlier (22).

### 2.6 Western blotting

Nuclear proteins were prepared using a nuclear extraction kit. The cells were treated as described in the legends and shown in the results at different times. Whole-cell lysates were obtained by resuspending the cell pellet in a lysis buffer containing protease and phosphatase inhibitors. Lysates were subjected to protein estimation by the Bradford method. Thereafter, western blotting was conducted as described earlier (22). Quantification of blots was done by using Image J software.

### 2.7 NF-κB DNA-binding activity assay

Breast cancer cells (1 × 10^5^) were grown in 6-well plates and exposed to TRX-01 (50 μM) at the indicated time points. The ability of NF-κB to interact with DNA were assayed using a commercial kit.

### 2.8 Statistical analysis

The student’s t-test detected the significance of differences between the control and TRX-01-treated groups. Each experiment was performed thrice and expressed as mean ± standard deviation.

## 3 Results

### 3.1 Density Functional Theory calculations

Gaussian09 was used to carry out computational DFT calculations for the TRX-01 compound (24). The Gaussview 5 software suite was utilized to construct and visualized. As seen in **Supplementary Figure S1 (Additional File)**, the TRX-01’s molecular shape was optimized at the B3LYP level using a 6–311++G (d,p) basis set (25).

### 3.2 Frontier Molecular Orbitals analysis

FMO (Frontier Molecular Orbital) analysis was conducted to assess the molecule’s kinetic stability and chemical reactivity using an appropriate basis set and method by DFT (26). The highest occupied molecular orbitals (HOMO) indicate electron donor capacity, whereas the lowest unoccupied molecular orbitals (LUMO) indicate electron acceptor ability, according to frontier molecular orbital theory (27). Consequently, examining the HOMO-LUMO energy levels offers valuable insights into the biological mechanism (28, 29). The energy gap is intimately correlated with the molecule’s kinetic stability and chemical reactivity (E_LUMO_-E_HOMO_). A smaller energy gap indicates lower stability and higher reactivity, while a larger one suggests greater chemical stability and lower chemical reactivity, indicative of increased hardness.

The computed values for HOMO and LUMO were found to be -6.231eV and -1.178eV, respectively. The calculated energy gap (E_LUMO_-E_HOMO_) of TRX-01 is 5.053 eV. Thus, the molecule under study is highly stable and less chemically reactive. It is observed from **Supplementary Figure S2 (Additional File)** that the HOMO of TRX-01 is localized in the region of 4-methoxy-2-((3-methylbenzyl) thio) pyrimidine and LUMO is delocalized in the 4-methoxypyrimidine-2-thiol region, especially on the sulfur atom and the pyrimidine ring of TRX-01. The global chemical reactivity descriptor parameters are computed based on the orbital energies, as shown in **Supplementary Table S1 (Additional File)**. The compound’s global hardness is 2.526 eV, which is high, with a lower softness value of 0.197 eV^-1^. Hence, the molecule has a low tendency to exchange its electron cloud. The chemical potential value is estimated at -3.704 eV, respectively, demonstrating their substantial ability to accept electrons. Strong electron-accepting capacity is indicated by a high chemical potential, whereas strong electron-donating ability is shown by a low chemical potential (27). It is determined that 1.256 eV is the electrophilicity index, which measures the energy required to stabilize a molecule.

### 3.3 Activation levels of NF‐κB signaling pathway in various breast cancer cell lines

**Figure 2:**
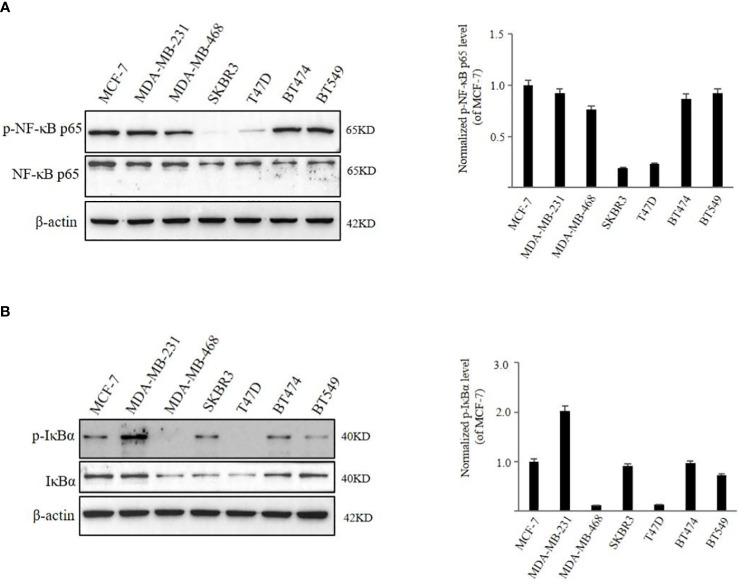
The activation level of NF‐κB signaling pathway in different breast cancer cell lines. **(A)** The expression of phospho‐NF-κB p65 and NF-κB p65 was detected by Western blot analysis. **(B)** The expression of p‐IκBα and IκBα was detected by Western blot analysis. Quantification of the values of p‐NF-κB p65 and p‐IκBα were normalized to NF-κB p65 and IκBα, respectively.

We first examined the expression of p-NF-κB p65 and NF-κB p65 in BC cells with different molecular phenotypes. We found that, in MCF-7, MDA-MB-231, MDA-MB-468, BT474, and BT549 cells higher expression of p-NF-κB p65 was observed in comparison to the levels in SKBR3 and T47D cells (**Figure 2A**). When NF-κB remains inactive, it forms a complex with IκBα and is localized within the cytoplasm. Upon phosphorylation of IκBα, NF-κB dissociates from IκBα, revealing its nuclear localization sequences. This allows NF-κB to translocate into the nucleus, facilitating NF-κB-dependent gene transcription. p-IκBα was found to be expressed in MCF-7, MDA-MB-231, SKBR3, BT474 cells and weakly expressed in BT549 cells. However, expression of p-IκBα was not detected in MDA-MB-468 and T47D cells (**Figure 2B**). Breast cancer cells are classified into several subtypes: Luminal A subtype, including MCF-7 and T47D cells; Luminal B subtype, such as BT474; HER-2 positive subtype, represented by SKBR3 cells; and triple-negative subtype, exemplified by MDA-MB-231, MDA-MB-468, and BT549 cells, based on their expression of ER, PR, HER-2, and Ki-67. To explore the impact of TRX-01 on various breast cancer subtypes, we selected cell lines with heightened NF-κB signaling pathway activation that also served as representatives of different subtypes for further investigation. Additionally, we assessed the activation level of the NF-κB signaling pathway in the normal breast cell line (MCF-10A), which exhibited significantly weaker activation compared to MCF-7, MDA-MB-231, and BT474 cells **Supplementary Figure S3 (Additional File)**.

### 3.4 TRX-01 reduces the viability of breast cancer cells

**Figure 3:**
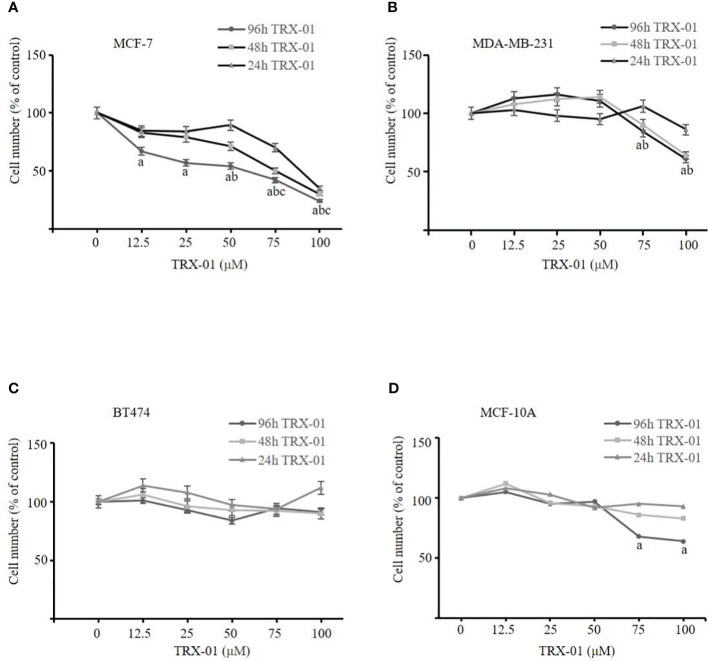
The effect of TRX-01 on the cell viability in breast cancer cells. **(A)** MCF‐7 cells were exposed to TRX-01 (0‐100 μM) for 24, 48, and 96 hours, and MTT analyzes cell proliferation. **(B)** MDA‐MB‐231 cells were exposed to TRX-01 (0‐100 μM) for 24, 48, and 96 hours, and MTT analyzed cell proliferation. **(C)** BT474 cells were exposed to TRX-01 (0‐100 μM) for 24, 48, and 96 hours, and MTT analyzed cell proliferation. **(D)** MCF-10A cells were exposed to TRX-01 (0‐100 μM) for 24, 48, and 96 hours, and MTT analyzed cell proliferation. Data are presented as mean ± standard deviation from three independent experiments. ^a^ *P* < 0.05 compared to the group without TRX-01 treatment for 96 hours; ^b^ *P* < 0.05 compared to the group without TRX-01 treatment for 48 hours; ^c^ *P* < 0.05 compared to the group without TRX-01 treatment for 24 hours. MTT, thiazolyl blue tetrazolium bromide.

**Figure 4:**
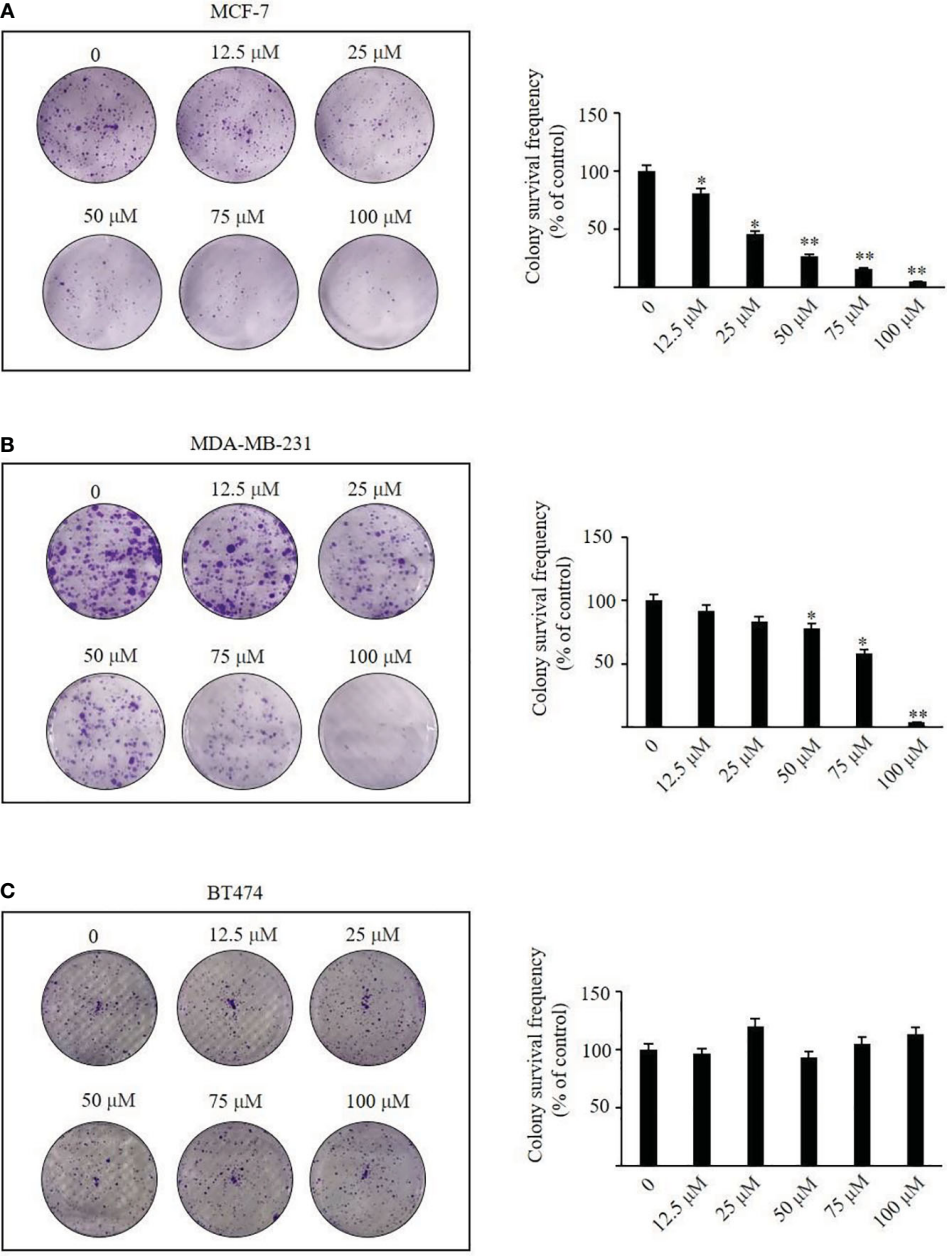
The effects of TRX-01 on colony formation were investigated in MCF-7, MDA-MB-231, and BT474 cells. After treatment with 0, 12.5, 25, 50, and 100 μM TRX-01 for 48 h, the colony formation was observed in MCF-7 cells **(A)**, MDA-MB-231 **(B)**, and BT474 **(C)**. (^*^ *P* < 0.05, ^**^ *P* < 0.01).

We administered TRX-01 at concentrations ranging from 0 to 100 μM to MCF-7, MDA-MB-231, and BT474 cells for periods between 24 and 96 hours and assessed cell viability through the MTT assay. The results indicated a significant reduction in the viability of MCF-7 cells, which was both dependent on the concentration of TRX-01 and the length of treatment time (**Figure 3A**). The half-maximal inhibitory concentration (IC50) of TRX-01 was found to be 53.9 μM. However, MDA‐MB‐231 and BT474 cells exhibited lower sensitivity to the drug than MCF-7 cells. A slight inhibitory effect on MDA‐MB‐231 cells was only seen when the drug concentration was increased to 75 μM (**Figure 3B**). Moreover, no inhibitory effect on BT474 cells was observed even when the drug concentration was increased to 100 μM (**Figure 3C**). To assess the impact of TRX-01 on normal breast epithelial cells, we opted for MCF-10A cells for our experiment. We observed that the proliferation of MCF-10A cells was only inhibited when the concentration of TRX-01 reached 75uM, (**Figure 3D**). This inhibition was significantly weaker compared to its effect on MCF-7 cells. We further performed colony formation experiments to confirm the effect of TRX-01 on cell viability. As shown in **Figure 4A**, TRX-01 reduced the percentage of surviving MCF-7 cell colonies in a concentration-dependent manner. As the concentration of TRX-01 was increased to 50 μM, the colony formation in MDA-MB-231 reduced (**Figure 4B**), while the colony formation in BT474 was not influenced by TRX-01 (**Figure 4C**).

### 3.5 TRX-01 suppresses cellular migration and invasion of breast cancer cells

**Figure 5:**
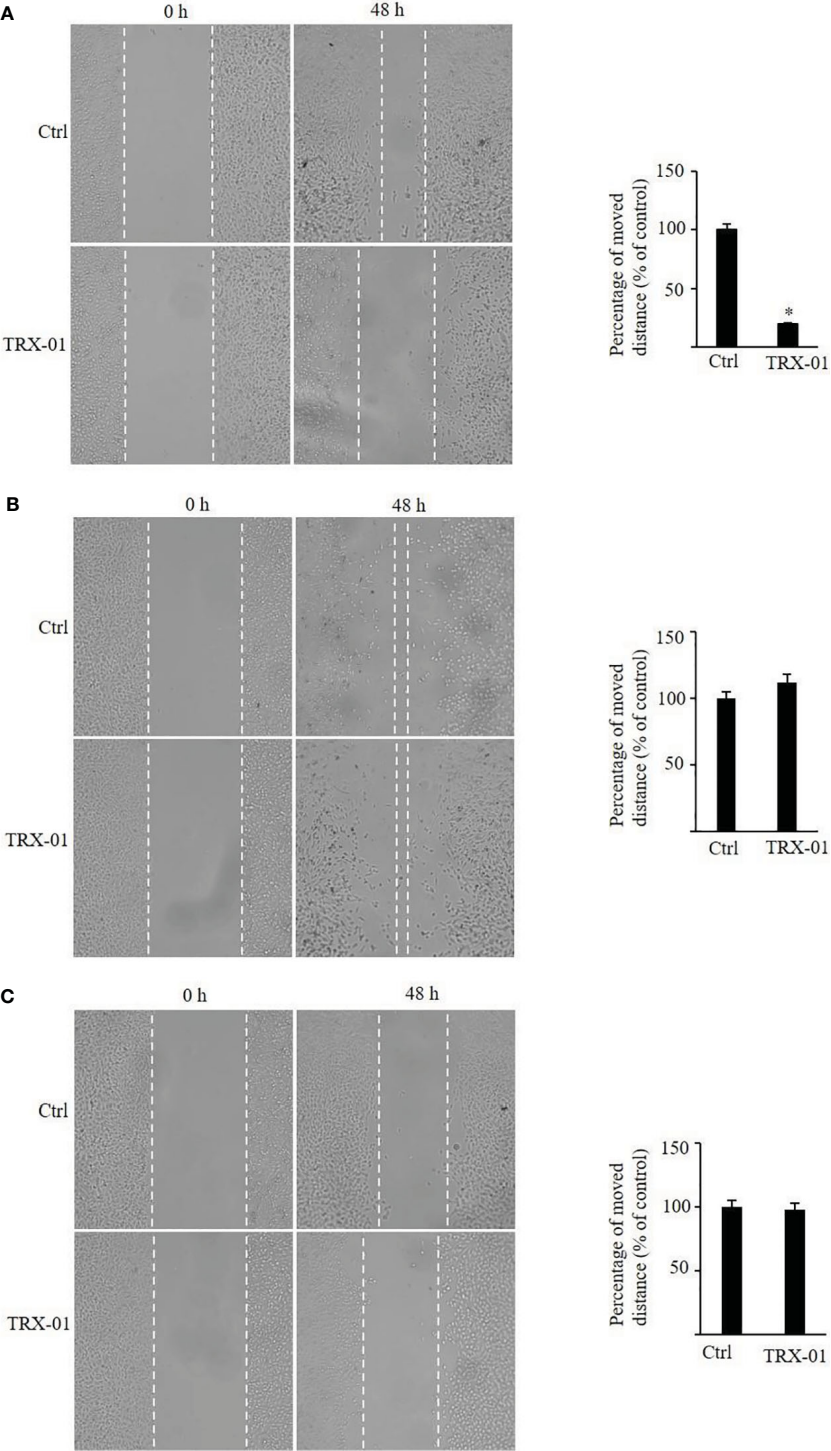
Effects of TRX-01 on the migration of breast cancer cells. **(A)** Scratch wound‐healing assay was performed in MCF-7 cells with or without TRX-01 (25 μM) treatment for 48 (h) **(B)**. Scratch wound‐healing assay was performed in MDA‐MB‐231 cells with or without TRX-01 (25 μM) treatment for 48 h. **(C)** Scratch wound‐healing assay was performed in BT474 cells with or without TRX-01 (25 μM) treatment for 48 (h) (^*^ *P* < 0.05).

**Figure 6:**
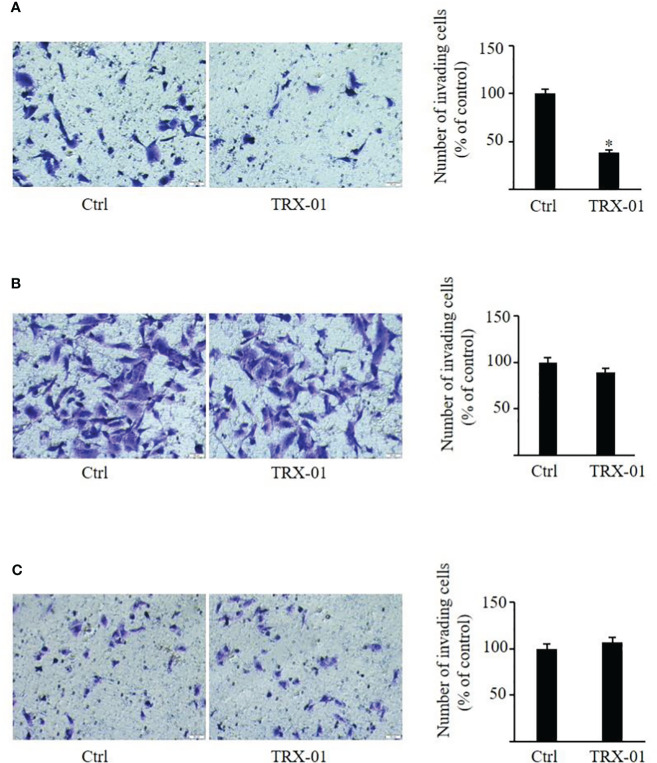
Effect of TRX-01 on the invasion of breast cancer cells. Invasion assays were performed in MCF-7 **(A)**, MDA-MB-231 **(B)**, and BT474 **(C)** cells with or without TRX-01 treatment (12.5 μM) for 72 (h) (^*^ *P* < 0.05).

The scratch wound healing was employed to analyze the impact of TRX-01 on the migration of MCF-7, MDA‐MB‐231 and BT474 cells. Various concentrations of TRX-01 (12.5 μM, 25 μM, and 50 μM) were tested in preliminary experiments. The findings revealed that treatment with 25 μM for 48 hours provided the optimal conditions for promoting scratch wound healing. We found that the migration of the MCF-7 cells was significantly inhibited upon TRX-01 treatment (**Figure 5A**). Meanwhile, TRX-01 did not suppress the migration of MDA‐MB‐231 and BT474 cells (**Figures 5B, C**). The effects of TRX-01 on the invasiveness of MCF-7, MDA-MB-231, and BT474 cells were evaluated using a chamber invasion assay. In the preliminary experiments, three concentrations of TRX-01 (12.5 μM, 25 μM, and 50 μM) were tested. The results indicated that a concentration of 12.5 μM, treated for 72 hours, yielded the most favorable conditions for the invasion assay. We observed that TRX-01 significantly reduced the invasiveness of MCF-7 cells (**Figure 6A**). However, TRX-01 did not significantly affect the invasiveness of MDA-MB-231 and BT474 cells (**Figures 6B, C**).

### 3.6 TRX-01 regulates the NF-κB signaling pathway in breast cancer cells

**Figure 7:**
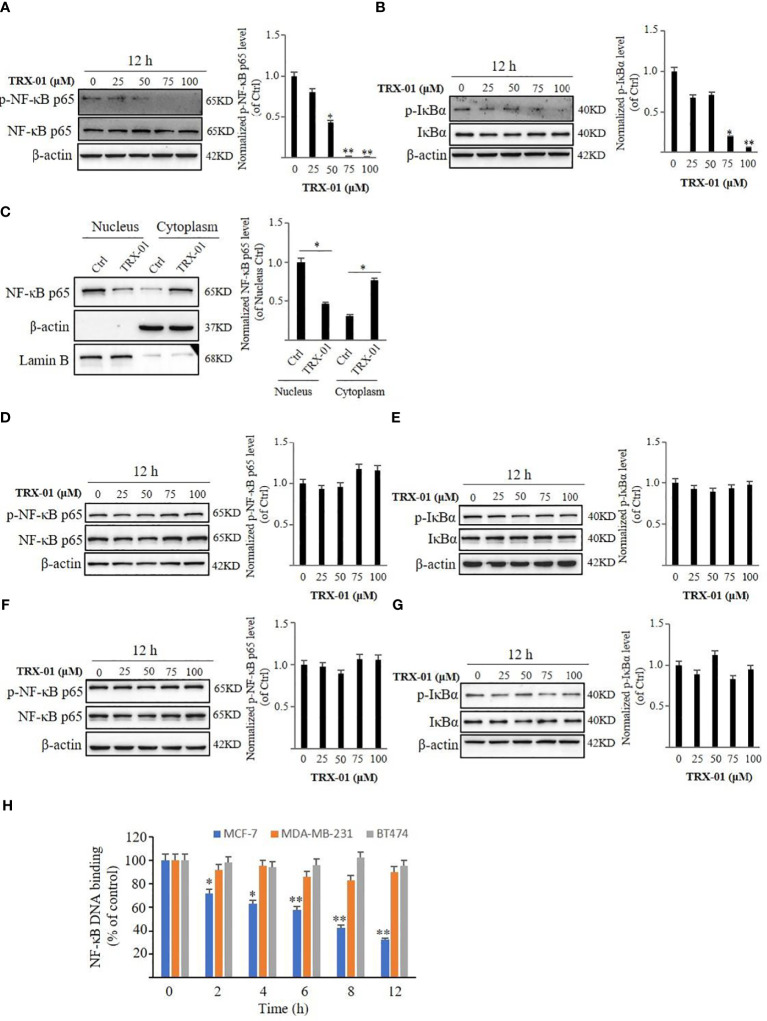
Effect of TRX-01 on NF‐κB activation in MCF-7, MDA-MB-231, and BT474 cells. MCF-7 cells were exposed to 0, 25, 50, 75, and 100 μM TRX-01 for 12 hours. The expression of phospho‐NF-κB p65 and NF-κB p65 **(A)** and the expression of p‐IκBα and IκBα **(B)** were detected by Western blot analysis. Moreover, the expression of NF-κB p65 in the nucleus and cytoplasm extract of MCF-7 cells with or without TRX-01 (50 μM) treatment for 12 hours was detected **(C)**. MDA-MB-231 cells were exposed to 0, 25, 50, 75, and 100 μM TRX-01 for 12 hours. The expression of phospho‐NF-κB p65 and NF-κB p65 **(D)** and the expression of p‐IκBα and IκBα **(E)** were detected by Western blot analysis. BT474 cells were exposed to 0, 25, 50, 75, and 100 μM TRX-01 for 12 hours. The expression of phospho‐NF-κB p65 and NF-κB p65 **(F)** and the expression of p‐IκBα and IκBα **(G)** were detected by Western blot analysis. Quantification of the values of p‐NF-κB p65 and p‐IκBα were normalized to NF-κB p65 and IκBα, respectively. **(H)** MCF-7, MDA-MB-231, and BT474 cells were treated with 50 μM TRX-01 for the duration. ELISA measured NF‐κB DNA binding activation. (^*^ *P* < 0.05, ^**^ *P* < 0.01).

The expression of the NF-κB pathway-related proteins, NF-κB p65 and p-NF-κB p65, were detected using Western blot analysis to determine the role of TRX-01 in the NF-κB pathway. TRX-01 decreased the expression of p-NF-κB p65 and p-IκBα in MCF-7 cells. However, the NF-κB p65 and IκBα protein levels were not markedly altered (**Figures 7A, B**). Western blot analysis was utilized to assess the expression levels of NF-κB p65 in the nuclear and cytoplasmic fractions of MCF-7 cells, treated with or without TRX-01, to further investigate how TRX-01 impacts the NF-κB signaling pathway. The findings revealed that TRX-01 notably suppressed the nuclear localization of NF-κB p65 in MCF-7 cells (**Figure 7C**), suggesting that TRX-01 disrupts the NF-κB pathway in these cells by blocking NF-κB p65’s movement into the nucleus. Additionally, the influence of TRX-01 on the NF-κB pathway was examined in MDA-MB-231 and BT474 cells. In MDA-MB-231 cells, the levels of phosphorylated NF-κB p65, phosphorylated IκBα, NF-κB p65, and IκBα remained largely unchanged (**Figures 7D, E**). The protein levels of p-NF-κB p65, p-IκBα, NF-κB p65, and IκBα in BT474 cells were also not affected by TRX-01 (**Figures 7F, G**). The interaction between NF-κB and DNA in MCF-7, MDA-MB-231, and BT474 cells, influenced by TRX-01, was measured using a commercial DNA binding kit. As shown in **Figure 7H**, the treatment with TRX-01 (50 μM) significantly inhibited the NF-κB activation in a duration-dependent manner in MCF-7 cells but not in MDA‐MB‐231 and BT474 cells.

### 3.7 TRX-01 attenuates the viability and mobility of MCF-7 cells by inhibiting the NF-κB signaling pathway

**Figure 8:**
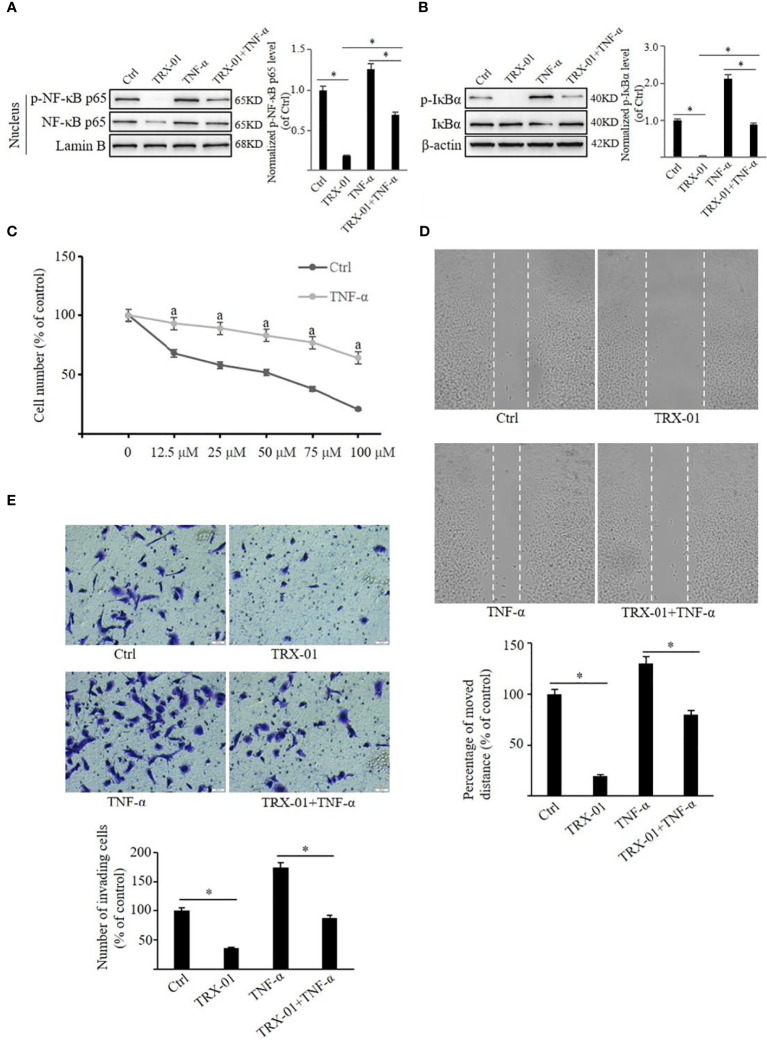
TRX-01 attenuates the growth and metastasis of MCF-7 by regulating the NF-κB signaling pathway. MCF-7 cells were treated with 50 μM TRX-01 for 12 h and then stimulated with 10 ng/ml TNF-α for 30 min to explore the role of the NF-κB signaling pathway in the effects of TRX-01 on breast cancer cells. Cells treated with DMSO were used as controls. The expression of phospho‐NF-κB p65 and NF-κB p65 in the nuclear extract **(A)** and the expression of p‐IκBα and IκBα in the whole cell extract **(B)** were detected by Western blot analysis. Quantification of the values of p‐NF-κB p65 and p‐IκBα were normalized to NF-κB p65 and IκBα, respectively. **(C)** MCF‐7 cells were exposed to TRX-01 (0‐100 μM) for 96 hours with or without TNF-α treatment (10 ng/ml), and MTT analyzes cell proliferation. ^a^ *P* < 0.05 compared to the group with TRX-01 treatment alone. **(D)** MCF‐7 cells were treated with or without 25 μM TRX-01 and 10 ng/ml TNF-α for 48 h and cell migration was assessed by Scratch wound‐healing assay. **(E)** MCF‐7 cells were treated with or without 12.5 μM TRX-01 and 10 ng/ml TNF-α for 72 h, and cell invasion was assessed by Transwell assays. (^*^ *P* < 0.05).

It was observed that NF-κB pathway was activated upon treatment with TNF-α. TNF-α increased p-NF-κB p65 and NF-κB p65 levels in the nucleus (**Figure 8A**). Similarly, the p-IκBα level was elevated by treatment of TNF-α, but no significant change in the expression level of IκBα was noted (**Figure 8B**). TRX-01 significantly attenuated TNF-α-mediated activation of the NF-κB pathway, as evidenced by a marked decrease in phosphorylation levels of IκBα and NF-κB p65 compared to the TNF-α group. In addition, administration of TNF-α significantly reduced the inhibitory effect of TRX-01 on MCF-7 cell proliferation (**Figure 8C**). Furthermore, it was observed that TNF-α-stimulated NF-κB signaling pathway activation abolished the inhibition of cell migration (**Figure 8D**) and invasion (**Figure 8E**) caused by TRX-01.

## 4 Discussion

Breast cancer is the most prevalent malignant tumor in women, posing a major risk leading to mortality. Breast cancer cells show high heterogeneity and can be categorized into different molecular phenotypes (Luminal A, Luminal B, HER-2 positive and triple-negative) based on ER, PR, HER-2, and Ki-67 (30). Luminal A breast cancer typically shows limited response to traditional chemotherapy drugs, with treatment primarily relying on endocrine therapy (31). However, in cases where patients with the luminal A subtype exhibit insensitivity or resistance to endocrine therapy, treatment outcomes can be unfavorable. Therefore, exploring additional therapeutic options becomes imperative in such scenarios (32). In this study, TRX-01 significantly inhibited the growth and motility of luminal A subtype breast cancer cells (MCF-7). In contrast, it had a poor inhibitory effect on triple-negative subtype (MDA-MB-231) and luminal B subtype (BT-474) breast cancer cells.

In a recent study, we had synthesized several oxazine-linked pyrimidines and piperazine-linked pyrimidine derivatives. We found that TRX-01, as an oxazine-linked pyrimidine, could inhibit the proliferation of breast cancer MCF-7 cells, and in silico docking studies predicted that it can bind the p65 subunit of NF–κB (19). Additionally, we found that oxazine-linked pyrimidine could inhibit the NF-κB signaling pathway in many tumor cells in our previous studies (10, 15, 16, 18, 19). In the present study, we first assessed the molecule’s kinetic stability and chemical reactivity of TRX-01 through bioinformatics and calculated the global chemical reactivity descriptor parameters of TRX-01. Thereafter, elevated levels of NF-κB activation were observed across various breast cancer cell lines, MCF-7, MDA-MB-231, and BT474 cells were specifically selected for further investigation. This decision was based on the identification of increased NF-κB activation and three representative subtypes of breast cancer cells.

In addition to the ER signaling pathway, numerous other signaling pathways within luminal A subtype breast cancer cells contribute significantly to biological behaviors such as proliferation, apoptosis, and migration. NF-κB stands out among these pathways as a pivotal player. In luminal A breast cancer, NF-κB activation can promote cell growth and survival, contributing to tumor progression and treatment resistance. Additionally, NF-κB signaling is implicated in orchestrating the complex interplay between cancer cells and their microenvironment, facilitating invasion and metastasis. Furthermore, NF-κB crosstalks with estrogen signaling pathways, enhancing the estrogen-driven proliferation of luminal A breast cancer cells. This interplay underscores the importance of NF-κB as a therapeutic target in this subtype of breast cancer (33). NF-κB primarily acts as a pro-inflammatory transcription factor that modulates the expression of various genes linked to inflammation, cell proliferation, metastasis, and apoptosis, playing a crucial role in regulating gene networks involved in tumorigenesis (34, 35). NF-κB family consists of five transcription factors: NF-κB1/p50, NF-κB2/p52, RelA/p65, RelB, and c-Rel. These components can form NF-κB complexes through either hetero- or homodimerization. In inactive cells, these components are predominantly located in the cytoplasm; upon activation, they translocate to the nucleus to initiate transcription. This process leads to the direct, indirect, or combined activation and regulation of multiple genes (36, 37). UV rays, cytokines like IL-2 and TNF-α, and antigens from bacteria and viruses activate NF-κB. The nuclear factor facilitates immune responses to infection, inflammation, cell division, and apoptosis (38). NF-κB activation occurs through two primary pathways: the canonical pathway and the non-canonical pathway. In the classical pathway, TNF-α binds to its receptor TNFR1, facilitated by the adaptor protein TNF-α receptor-associated death domain (TRADD). This interaction is linked to both the TNF receptor-associated factor (TRAF) and the receptor-interacting protein (RIP). This causes activation of the IKK complex. When activated, IκB is phosphorylated and then degraded, which allows the p50-p65 subunits to enter into the nucleus, stimulating the transcriptional function of NF-κB (39, 40).

Pharmacological inhibition of the acetyl-lysine reader, cat eye syndrome chromosome region candidate 2 (CECR2), has been found to impede macrophage-mediated NF-κB immunosuppression and to inhibit breast cancer metastasis (41). Previous findings suggest that heat shock protein beta-1 (HSPB1) contributes to doxorubicin resistance by shielding breast cancer cells from drug-induced ferroptosis. Through mechanistic investigations, it was found that HSPB1 interacts with IκBα, facilitating its ubiquitination and subsequent degradation. This process leads to enhanced nuclear translocation and activation of the NF-κB signaling pathway. These discoveries imply that HSPB1 fosters chemoresistance by preventing ferroptosis and influencing the NF-κB signaling pathway in breast cancer cells. The elevation of HSPB1 levels could be a significant factor in the advancement of breast cancer and its resistance to chemotherapy (42). Moreover, the joining chain of multimeric IgA and IgM (JCHAIN) has been identified as capable of hindering the growth and mobility of breast cancer cells by suppressing NF-κB -mediated EMT (43). Recently, we also found that some compounds could promote apoptosis and inhibit cell proliferation as well as migration ability by inhibiting NF-κB signaling pathway in triple-negative breast cancer cells (44). All these evidences have proved that NF-κB plays an important role in the development of breast cancer cells. This study found that TRX-01 could inhibit the phosphorylation of NF-κB p65 and IκBα in MCF-7 cells in a concentration-dependent manner. However, it did not have substantial effect on the total amount of NF-κB p65 and IκBα in MCF-7 cells. Further analysis involving the extraction of cytoplasmic and nuclear proteins to assess NF-κB p65 levels indicated a shift in the distribution of NF-κB p65 levels, confirming TRX-01’s capability to block the nuclear entry of NF-κB p65, thereby inhibiting the expression of target genes. However, the precise mechanisms through which these effects are achieved warrant further detailed investigation. TRX-01 showed no significant influence on the NF-κB signaling pathway in MDA-MB-231 and BT474 cells. Additionally, the study revealed that TRX-01 could suppress the DNA-binding activity of NF-κB in MCF-7 cells. To verify whether TRX-01’s suppression of MCF-7 cell proliferation and motility was through inhibiting the NF-κB signaling pathway, TNF-α was employed to activate the pathway. The findings indicated that TNF-α could partially mitigate the suppressive effects of TRX-01 on the proliferation and motility of MCF-7 cells.

This report demonstrated the detailed effects of TRX-01 on breast cancer cells with different subtypes, confirming its unique effect on luminal A subtype breast cancer cells. Although it was found that the inhibitory effect of TRX-01 on MCF-7 cells was accomplished by blocking NF-ĸB signaling pathway, further experiments are needed to comprehensively understand how TRX-01 suppresses the NF-κB signaling pathway in luminal A subtype breast cancer cells. This could involve studying its interaction with specific NF-κB components and downstream effectors. In addition, preclinical studies using various breast cancer models, including cell line xenografts and patient-derived xenografts (PDX), can provide valuable insights into the efficacy and mechanism of action of TRX-01 *in vivo*. Finally, future studies could focus on exploring potential synergistic effects of TRX-01 in combination with existing endocrine therapies or other targeted agents. This could lead to the development of more effective treatment regimens for luminal A breast cancer.

## 5 Conclusions

TRX-01 inhibited the growth and motility of luminal A subtype breast cancer cells by suppressing the NF-κB signaling pathway. These findings could offer a novel alternative to endocrine therapy for treating luminal A subtype breast cancer. Furthermore, future research could evaluate the efficacy of TRX-01 in luminal A subtype breast cancer cells that have developed resistance to endocrine therapy. If TRX-01 demonstrates comparable sensitivity in these resistant cells as it does in their endocrine therapy-sensitive counterparts, it could represent a significant advancement in addressing the challenge of endocrine therapy resistance. This promising prospect underscores the potential value of TRX-01 as a supplementary treatment option in the fight against breast cancer, especially for patients who have become unresponsive to conventional endocrine therapies.

## Data availability statement

The datasets presented in this article are not readily available. Requests to access the datasets should be directed to dr.yuanjie@outlook.com.

## Author contributions

JY: Writing – original draft, Investigation. BN: Writing – review & editing, Formal analysis, Data curation, Conceptualization. AR: Writing – original draft, Investigation, Data curation. LY: Writing – review & editing, Data curation. HZ: Writing – review & editing, Data curation, Conceptualization. QW: Writing – review & editing, Formal analysis. ZL: Writing – review & editing, Data curation. JH: Writing – review & editing, Investigation, Formal analysis. BW: Writing – review & editing, Resources, Methodology. GW: Writing – review & editing, Supervision, Funding acquisition. KK: Writing – review & editing, Resources, Methodology. AC: Writing – review & editing, Supervision, Methodology. CG: Writing – review & editing, Resources, Methodology. MM: Writing – review & editing, Methodology, Formal Analysis. BB: Writing – review & editing, Validation, Supervision, Resources.
