# Supplementary material for: Discovery of oxazine-linked pyrimidine as an inhibitor of breast cancer growth and metastasis by abrogating NF-κB activation
Source: Front Oncol. 2024 Jul 29;14:1390992. doi: 10.3389/fonc.2024.1390992 (PMC11317417; doi:10.3389/fonc.2024.1390992)
Supplement: Supplementary file 1 [file DataSheet_1.docx]

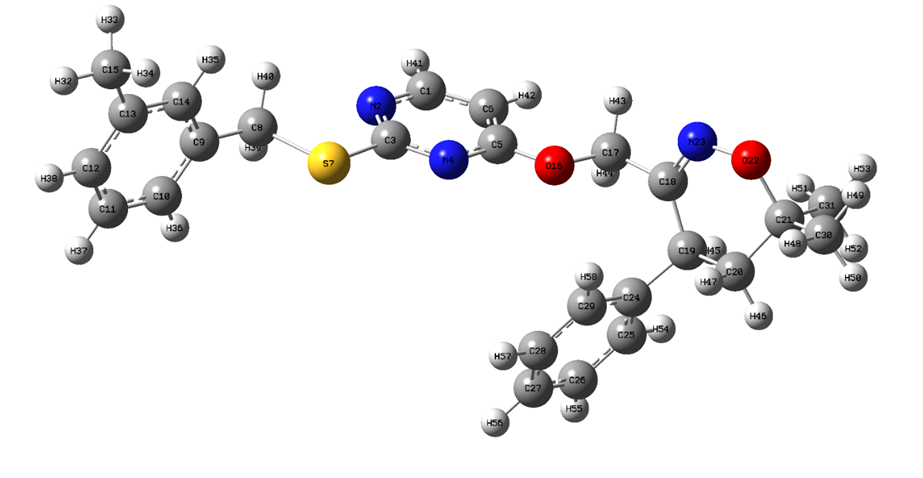

**Figure S1.** Optimized geometry of TRX-01 using B3LYP 6311++G (d, p) method by density functional theory.


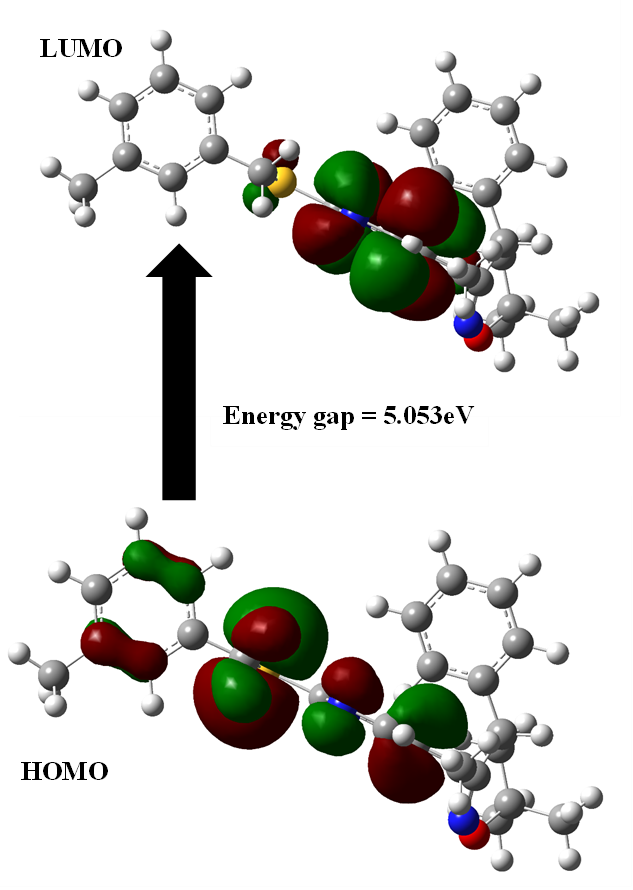


**Figure S2.** Frontier molecular orbital plots of TRX-01.


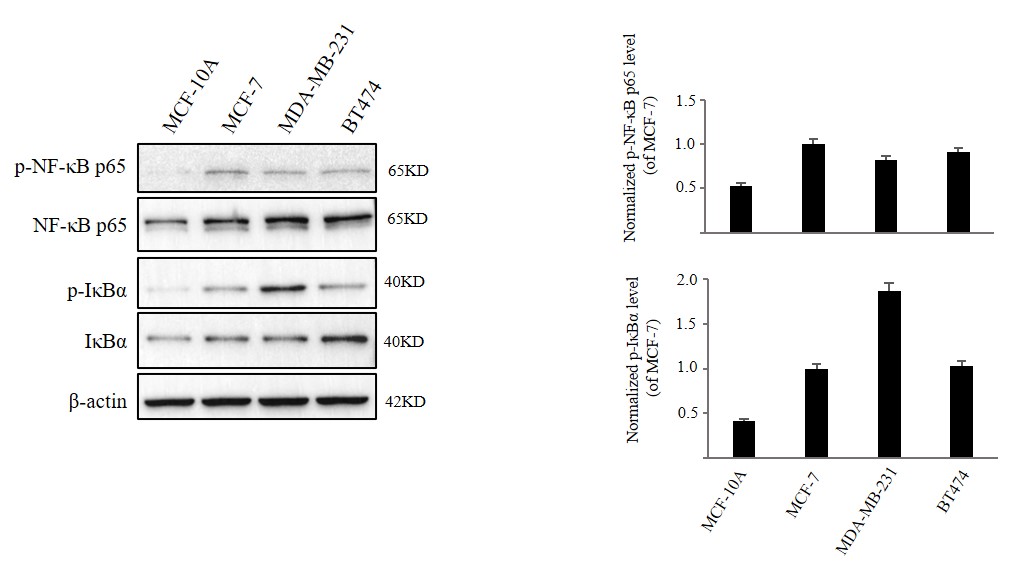


**Figure S3. The activation level of NF‐κB in the normal breast cell and few breast cancer cell lines.** The expressions of p‐NF-κB p65, NF-κB p65, p‐IκBα, and IκBα were detected in MCF-10A cells, MCF-7, MDA-MB-231, and BT474 cells. Quantifications of the values of p‐NF-κB p65 and p‐IκBα were normalized to NF-κB p65 and IκBα, respectively.

**Table S1.** Global Chemical Reactivity Descriptors of TRX-01.

| **Molecule Properties** | **TRX-01** |
| --- | --- |
| Energy in a. u. | -1682.124 |
| E_HOMO_ in eV | -6.231 |
| E_LOMO_ in eV | -1.178 |
| E_HOMO-1_ in eV | -6.691 |
| E_LUMO+1_ in eV | -0.894 |
| ∆E_LUMO-HOMO_ in eV | 5.053 |
| Ionization potential(I) | 6.231 |
| Electron affinity (A) | 1.178 |
| Global Hardness (ɳ) | 2.526 |
| Softness (S) | 0.197 |
| Chemical potential (μ) | -3.704 |
| Electronegativity (χ) | 3.704 |
| Electrophilicity (ᴪ) | 1.256 |
| Dipole moment in Debye | 4.315 |
